# Impact of Tau on Neurovascular Pathology in Alzheimer's Disease

**DOI:** 10.3389/fneur.2020.573324

**Published:** 2021-01-07

**Authors:** Elisa Canepa, Silvia Fossati

**Affiliations:** Alzheimer's Center at Temple (ACT), Lewis Katz School of Medicine, Temple University, Philadelphia, PA, United States

**Keywords:** tau, neurovascular unit, mitochondria, caspases, Alzheimer's disease, tauopathies, vascular dysfunction

## Abstract

Alzheimer's disease (AD) is a chronic neurodegenerative disorder and the most prevalent cause of dementia. The main cerebral histological hallmarks are represented by parenchymal insoluble deposits of amyloid beta (Aβ plaques) and neurofibrillary tangles (NFT), intracellular filamentous inclusions of tau, a microtubule-associated protein. It is well-established that cerebrovascular dysfunction is an early feature of AD pathology, but the detrimental mechanisms leading to blood vessel impairment and the associated neurovascular deregulation are not fully understood. In 90% of AD cases, Aβ deposition around the brain vasculature, known as cerebral amyloid angiopathy (CAA), alters blood brain barrier (BBB) essential functions. While the effects of vascular Aβ accumulation are better documented, the scientific community has only recently started to consider the impact of tau on neurovascular pathology in AD. Emerging compelling evidence points to transmission of neuronal tau to different brain cells, including astrocytes, as well as to the release of tau into brain interstitial fluids, which may lead to perivascular neurofibrillar tau accumulation and toxicity, affecting vessel architecture, cerebral blood flow (CBF), and vascular permeability. BBB integrity and functionality may therefore be impacted by pathological tau, consequentially accelerating the progression of the disease. Tau aggregates have also been shown to induce mitochondrial damage: it is known that tau impairs mitochondrial localization, distribution and dynamics, alters ATP and reactive oxygen species production, and compromises oxidative phosphorylation systems. In light of this previous knowledge, we postulate that tau can initiate neurovascular pathology in AD through mitochondrial dysregulation. In this review, we will explore the literature investigating tau pathology contribution to the malfunction of the brain vasculature and neurovascular unit, and its association with mitochondrial alterations and caspase activation, in cellular, animal, and human studies of AD and tauopathies.

## Introduction

### Alzheimer's Disease, Neurovascular Unit and Blood-Brain Barrier Dysfunction

Alzheimer's disease (AD) is a progressive and deadly neurodegenerative disorder recognized by the World Health Organization as the most prevalent form of dementia (1). AD initial symptoms typically manifest as mild cognitive impairment (MCI). Although MCI does not always convert to AD or dementia, a percentage of MCI cases will worsen over the years, eventually progressing to the severe cognitive decline, emotional and behavioral changes, visuospatial and motor deficits, characteristics of late-stage AD (2). These devastating cognitive and physical aspects are correlated with neuronal loss and extensive brain atrophy, predominantly in the hippocampus and cortex (3). The most studied and scientifically confirmed neuropathological hallmarks of AD are senile plaques (SP) and neurofibrillary tangles (NFT) in association with neuronal degeneration, loss of synapses, neuroinflammation, and oxidative stress (4). Furthermore, conspicuous studies indicate that brain vascular dysregulation is an early feature of AD, contributing to the progression of the pathology, and suggesting a tight link between cerebrovascular alterations and neurodegeneration (5–8). SP are abnormal parenchymal insoluble deposits of amyloid-beta (Aβ), which derives from amyloid precursor protein (APP) processing. Aβ monomers aggregate into more complex species (oligomers and protofibrils) which represent the toxic species for most brain cells (9, 10) and eventually form fibrils (11). Interestingly, Aβ accumulation also takes place around the brain micro- and macro-vasculature, in a well-described AD pathological feature known as cerebral amyloid angiopathy (CAA) (12), which leads to severe cerebral vascular dysfunction (CVD) (10, 13–16). NFT are intracellular inclusions of paired helical filaments (PHF) of the microtubule-associated protein (MAP) tau. Physiologically, specific phosphorylation and dephosphorylation events regulate tau microtubule (MT)-binding property, thus modulating tau functions, including promoting MT assembly and dynamics, and maintaining neuronal spatial organization and stability (17, 18). In AD brains, tau phosphorylation levels are 3–4-fold higher compared to non-demented adult brains (19, 20), and phosphorylation facilitates tau aggregation, causing cell morphology, functionality, and viability disruption. Besides the documented effects of Aβ deposition around cerebral blood vessels (BVs), recent evidence indicates that tau pathology induces CVD (21–23), and that the presence of hippocampal perivascular tau strictly correlates with blood brain barrier (BBB) permeability and loss of integrity (24), prompting new questions to elucidate the molecular mechanisms through which tau toxicity affects the cerebral vasculature, in both AD and other tauopathies.

The BBB is a highly specialized and selective semipermeable interface between the central nervous system (CNS) and the peripheral circulation, regulating the entrance of blood specific components into the brain and the clearance of potentially neurotoxic substances from the CNS to the blood. The morpho-functional unit, which includes the BBB and is responsible for maintaining its unique properties, is the neurovascular unit (NVU). The NVU is constituted by endothelial cells (ECs) that form the BVs and are strongly attached to each other via a complex network of sealing proteins named tight junctions (TJs) (25), by the surrounding smooth muscle cells (SMC) or pericytes, together with neurons, astrocytes, and microglia cells (8, 26–28). BV-surrounding cells influence and co-operate in maintaining the structural and functional BBB phenotype (26). Astrocytes are the major glial cell type (~98%) at the parenchymal basal membrane of the microvasculature (29), with microglial cells occupying the remaining surface. In addition to their contribution to BBB maintenance and permeability, astrocytes are considered immunocompetent cells, playing a central role in detecting detrimental signals following which they start secreting cytokines and chemokines, boosting innate and adaptive immune cell activation and trafficking, and thus affecting BBB function (30). Microglia are brain innate resident immune cells, involved in the active surveillance of CNS. When exposed to pathogens, tissue damage or toxic substances, microglial cells become activated, releasing pro-inflammatory cytokines, chemotactic factors, and mediate adaptive immunity, acting as antigen-presenting cells (31, 32). If activated, they can interact with cerebral microvasculature, and increase capillary permeability by producing reactive oxygen species (ROS), and promoting monocyte and lymphocyte migration through the BBB (33, 34). Importantly, the extensive astrogliosis and microgliosis present in AD brains (35–38), may further exacerbate vascular dysfunction. Morphologically, AD brains display BBB changes such as reduced microvascular density, increased capillary tortuosity and fragmentation with fewer intact branches, atrophic string vessels, and changes in vessel diameter (8, 39, 40), that impair vascular fitness causing decreased cerebral blood flow (CBF), hypoxia, dysregulated nutrient and oxygen transport to the CNS, and disrupted cerebral clearance. As a consequence, amplification of cellular stress, accumulation of toxic metabolic waste, uncontrolled detrimental inflammatory response and infiltration of blood-borne molecules and cells occur, eliciting neurodegenerative processes and progressive decrease in cognitive functions (8, 41, 42). All together, these data provide a strong evidence of the connection between BBB malfunction and AD, although the leading molecular mechanisms triggering CVD have not been fully elucidated.

While Aβ aggregation and deposition have long been associated with cerebrovascular alterations in AD, the scientific community has recently started to consider the role of tau in BBB dysfunction, supported by a rapidly growing literature that confirms CVD in other tauopathies.

Here, we will review what is known about tau and neurovascular dysregulation, exploring and providing perspective on some of the possible molecular events through which tau may exert its toxicity on the NVU.

### Tau and Its Transmission

The MAPs family includes three classes of polypeptides predominantly expressed in neurons: MAP1, MAP2, and tau. While MAP1 and MAP2 have been largely found in dendrites, tau has been detected mainly in axons (43, 44), where it is involved in primary functions, including neuronal development control (45–47), vesicular and axonal transport (48, 49), and neuronal polarity maintenance (50), amongst others. Structurally, tau presents a basic proline-rich region (aa155–242) which contains serine (S), threonine (T), and tyrosine (Y) potential phosphorylation sites (51). In physiological state, tau is phosphorylated or dephosphorylated based on the balance of kinase (e.g., GSK3β and CDK5) and phosphatase (e.g., PP1, PP2A, B and C) activities (47, 52–55). This equilibrium confers tau the ability to bind and stabilize tubulin polymerization, requisite condition for maintaining axonal and dendritic shape, and thus functionality (56, 57). Due to hyperphosphorylation, tau MT-binding property is lost, inducing its oligomerization and the formation of PHF which progressively aggregate into NFT (58, 59). As a consequence, alterations of cytoskeleton architecture occur, leading to axonal transport disruption, synaptic dysfunction, and eventually neuronal cell death (47, 60–62). Indeed, a characteristic brain accumulation of highly phosphorylated tau is found in AD, and other tauopathies, such as progressive supranuclear palsy (PSP), Pick's disease (PiD), corticobasal degeneration (CBD), and frontotemporal dementia FTD (63).

Although research in neurodegenerative disorders focuses mainly on tau hyperphosphorylation, it is relevant to mention that tau can also be subject to concurrent or alternative post-translational modifications (PTMs), including N- and C-terminal proteolytic cleavage (truncation), nitration, glycosylation, acetylation, glycation, ubiquitination, or polyamination (64), that exacerbate tau pathology. For instance, in AD human brains, C-terminal tau fragments generated by caspase-3 cleavage at aspartic acid residue 421 (D421) have been detected (65). Considerable evidence shows that caspase-3-truncated tau species are particularly prone to phosphorylation (65), and that caspase-dependent cleavage process increases tau propensity to self-aggregate, boosting the rate of tau polymerization and NFT assembly (45, 47, 59, 66), and fostering dendritic spine loss, synaptic impairment and memory deficits (67, 68). Similarly, caspase-6 activity derived N-terminal truncated tau segments have been found in AD cerebral tissue (69).

Collectively, these data indicate that misfolded conformations of tau facilitate the development of aggregates that appear to be crucial for neuronal demise in AD and other tauopathies.

In the last decade, a wealth of studies has suggested that misfolded tau spreads in a prion-like manner in tauopathy brains (70–72). The hypothesis of the prion-like propagation has been proposed from the observation that the progressive accumulation of tau appeared to spread in a foreseeable pattern, along known anatomically connected neuronal networks (73), analogously to that reported for prion proteins (72).

**Figure 1:**
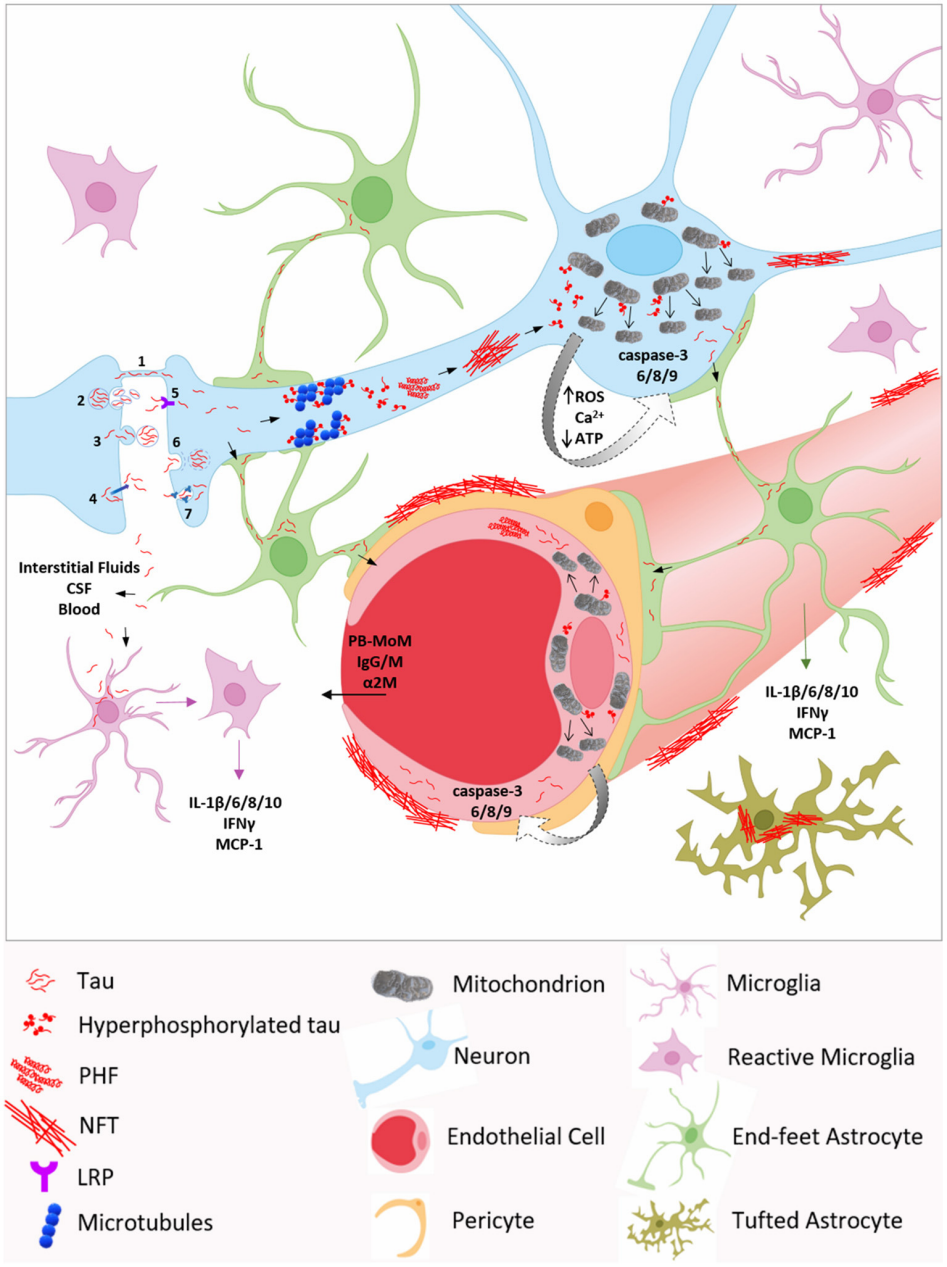
Molecular mechanisms underlying neurovascular tau toxicity in AD and other tauopathies. Neuronal transmission through (1) tunneling nanotubes (TNTs), (2) exosomes, (3) ectosomes, or (4) plasma membrane may account for extracellular tau, which can be internalized by neighboring neurons *via* (5) low-density lipoprotein receptor (LRP), (6) clathrin-mediated endocytosis, (7) micropinocytosis by heparin sulfate proteoglycans, or (1) TNTs. Tau is also detected in brain interstitial fluids, cerebrospinal fluid (CSF) and in blood, reflecting the intensity of neurodegeneration. Inside the neuron, hyperphosphorylated tau promotes microtubules disassembly, aggregates into oligomers and paired helical filaments (PHF) which, in turn, accumulate, leading to neurofibrillary tangles (NFT) deposition. Phosphorylated tau can interact with mitochondrial proteins, such as Drp1, triggering excessive mitochondrial fission and mitochondrial dysfunction, including elevated ROS production, Ca^2+^ homeostasis dysregulation, decreased ATP production, and ultimately caspase activation. Caspase-3 cleaved tau facilitates tau phosphorylation and self-aggregation, further exacerbating tau pathology. Tau can propagate to astrocytes and microglia, and be internalized *via* micropinocytosis and CX3CR1, respectively. In astrocytes, the accumulation of tau fibrils around the nucleus confers the characteristic tufted phenotype. Tau pathological species, through astrocytic end-feet and interstitial fluids, may spread to endothelial cells and pericytes, inducing blood brain barrier (BBB) disruption and permeability to blood-borne components, including peripheral blood monocyte-derived macrophages (PB-MoM), immunoglubulins (IgGs and IgMs), and α2-macroglobulin (α2M). Moreover, the presence of tau within glial cells induces the secretion of several pro-inflammatory cytokines and chemokines, such as IL-1β,−6, - 8,−10, IFNγ, and MCP-1, initiating a high inflammatory state, which contributes to BBB integrity loss.

Albeit tau is an intracellular protein, both tau and P-tau can also be detected in brain interstitial fluid (74), cerebrospinal fluid (CSF) (75), and, as shown more recently, in blood (76–79). In AD patients, CSF and plasma tau and phosphorylated tau (P-tau) concentrations are significantly higher compared to healthy controls. Since tau in biofluids is considered to be for the most part neuron-derived, CSF and plasma tau concentrations typically represent the intensity of neurodegeneration (76, 78, 80, 81). CSF P-tau, on the other hand, is an established AD biomarker also used in clinical trials, reflecting more specifically the progression of tau pathology and correlating with cognitive dysfunction. Recently, plasma P-tau^181^ and P-tau^217^ have also been proposed as novel biomarkers for AD (82–84). Although blood tau and P-tau fragments are of great interest as biomarkers, these peptides are present at very low concentrations in the circulation (in the low pg/ml), and it is still unknown if circulating tau species may be able to induce toxic effects on the vasculature, both in the brain and in the periphery. In particular, more studies are needed to clarify any possible toxic effects of blood P-tau, especially in conditions in which cerebral vessels are already dysfunctional. On the other hand, CSF P-tau, present at higher concentrations in the perivascular spaces, may be a likely contributor to neurovascular pathology, especially in situations in which perivascular clearance is compromised, which may facilitate its accumulation around brain vessels (85–87). Interestingly, it has been reported that, besides prompting intracellular toxicity, tau can be secreted and taken up by healthy neurons (88–90), indicating that extracellular or CSF P-tau may be critical for the progression of tau pathology. Recent studies in the AD field have demonstrated that seed-competent tau spreads along neural connections, is detected in synaptosomes and white matter axons, and that tau seeding precedes the presence of hyperphosphorylated tau in synaptically connected regions (91–94). To further elucidate the propagating properties of tau, many studies have investigated the potential molecular processes involved. Numerous neuronal mechanisms have been identified as non-mutually exclusive sources of extracellular tau, including synaptic secretion (95), direct unconventional translocation across the plasma-membrane (96, 97), release in extracellular vesicles such as exosomes (98–100) and ectosomes (101), or tunneling nanotubes (TNTs) (102, 103). In pathological conditions, neuronal deterioration may also account for extracellular tau which is internalized by neighboring cells via bulk endocytosis (88, 89, 104, 105), clathrin- (106, 107) or low-density lipoprotein receptor related protein 1 (LRP1)-mediated endocytosis (108), micropinocytosis by heparin sulfate proteoglycans (90, 109–111) or TNTs (102, 103) (Figure 1). Following cellular entry of tau, neurons can seed physiological monomers, perpetuating the pathological process (112). It has also been demonstrated that neuronal activity can induce tau release (95), enhancing intercellular spread of tau (113).

As mentioned above, AD and tauopathy brains display widespread astrogliosis and microgliosis, which closely correlate with the areas of neurodegeneration (114, 115). Intriguingly, several studies have reported the accumulation of toxic tau in astrocytes and microglia in different tauopathies (116–121). Endogenous glial tau expression has been reported (122, 123), although at much lower levels compared to neurons (124). Therefore, it has been postulated that the intracellular presence of tau in these cell types may be, at least in part, explained by its internalization from the extracellular milieu. Recent evidence has demonstrated that astrocytic tau uptake occurs via micropinocytosis, followed by enhanced lysosomal pathways (125). It is likely that, in pathological conditions, tau engulfed astrocytes exhibit dysregulated clearance mechanisms, further intensifying tau propagation and toxicity. Additionally, it is known that microglia have the competence to phagocytize tau (126–132), and the presence of microglial aggregated or hyperphosphorylated tau has been observed in mice ad humans with tau pathology (119, 120, 126–128, 131, 133). The microglial process of tau internalization seems to be mediated by CX3CR1 receptor (120, 133). More importantly, at the site of tau colocalization with astrocytes and microglia, several pro-inflammatory cytokines have been detected (134), indicating a high neuroinflammatory state. During inflammation, reactive glial cells continuously secrete cytokines and chemokines, which further recruit and activate innate and adaptive immune cells (30–32), initiating a feed-forward detrimental inflammatory response, feature of AD and tauopathies (4, 134), which may also affects neurovascular and BBB function. Another hypothesis proposes that tau toxicity might be mediated by the brain vasculature, considering that vascular dysfunction, dendritic deterioration and inflammation take place before neuronal loss in a mouse model of tau-induced neurodegeneration (135) or before tau phosphorylation in a salt-rich diet model (136). In turn, tau toxicity may also be in part responsible for the vascular dysfunction present in AD and related disorders. In confirmation of this, considerable studies have shown that tau pathology triggers alterations of BVs and vascular inflammation (23, 135, 137–139), which may be induced by tau oligomers and fibrils deposition around the microvasculature (23, 24, 140). Moreover, tau-induced neuroinflammation can additionally damage BBB, leading to the infiltration of peripheral immune cells and endothelial signaling molecules expression (141, 142), which keep glial cells active (22, 143), worsening inflammation, neurovascular pathology, and consequentially neuronal activity.

All together, these results suggest how tau toxicity may propagate from neurons to NVU components, and potentially activate harmful mechanisms that interfere with neurovascular function and inflammation, exacerbating neurodegeneration.

## TAU and Neurovascular Dysfunction

### Cell Studies

As previously described, the BBB is a dynamic and extremely specialized barrier which physically separates CNS and systemic circulation. The NVU is a complex functional unit including the BBB and is responsible for its properties and health. ECs, mural cells (including vascular smooth muscle cells and pericytes), astrocytes, microglia and neurons (26, 144) compose the NVU and contribute to its functions. Multiple studies have confirmed NVU abnormalities in AD, describing CVD as an early event in AD pathology, and establishing a direct correlation between brain vasculature dysregulation and neurodegeneration (5–8). The potential molecular pathways linking tau toxicity and neurovascular dysfunction have recently started to be evaluated, both *in vitro* and *in vivo* (Table 1). As reported below, a growing body of literature shows that other tauopathies, in addition to AD, also display severe cerebrovascular changes (22, 24, 28, 142, 145–149) (Table 2).

**Table 1:** Effects of tau on the NVU *in vitro* and *in vivo* models.

***In vivo*/*in vitro* model**	**Tau isoform**	**Effects**	**References**
RBEC (primary rat brain ECs)	Human oligomeric tau	Increased expression of genes related to inflammation, endocytosis, angiogenesis, blood coagulation, vasoconstriction, and diapedesis. Accelerated peripheral blood monocyte-derived macrophages transmigration, when RBEC are exposed to conditioned media of astrocytes pre-treated with tau. Reduced peripheral blood monocyte-derived macrophages transmigration, when RBEC are treated with antibodies anti-ICAM1 and anti-VCAM1.	(142)
co-culture RBEC + primary rat astrocytes and microglia	Human truncated tau	RBEC show decreased TEER and augmented mannitol permeability, when tau is added to the abluminal (glial) side. Glial cells increase TNF-α and MCP-1 release.	(22)
rat microglia	Human tau40	Higher rate of migration, enhanced phagocytosis and release of NO, IL-6, IL-1β, TNF-α, and IL-10.	(145)
WT FVB/NJ mouse	AAV-P310L tau injection (neuronal expression)	10 days after cortical injection, capillary thickness is increased, and hippocampal BVs are surrounded by swollen astrocytes. 3–6 weeks following injection, neuronal loss, microgliosis and IgGs, IgMs, and α2-macroglobulin presence in cerebral parenchyma.	(135)
Tg4510 mouse	P310L tau (neuronal expression)	Perivascular tau accumulation. CD3^+^ and CD4^+^ lymphocytes, and RBC infiltration along brain vasculature. Extravasation of IgGs and Evans Blue, mainly in the hippocampus and cortex. Glial activation. BBB functional recovery following doxycycline treatment (which suppresses tau expression).	(24)
Tg SHR-72 rat	Human truncated tau (neuronal expression)	In brainstem, peripheral blood monocyte-derived macrophages transmigration and increased ICAM-1 expression.	(142)
Tg4510 mouse	P310L tau (neuronal expression)	Increased number of capillaries with atypical and spiraling morphologies. Reduced BV diameter and elevated cortical BV density. Altered expression of hypoxia- and angiogenesis-related genes in both ECs and microglia.	(137)
Tg GFAP/tau mouse	T34 human tau (astrocytic expression)	IgGs and albumin presence in brain parenchyma. Tau pathology in astrocytes surrounding BVs.	(146)

**Table 2:** Effects of tau on the NVU in AD and other tauopathies.

**Disease**	**Tau isoform**	**Effects**	**References**
Alzheimer's disease	NFT	Small and medium size artery SMC loss occurs between early onset tau toxicity-Braak stage I and II–III, along with PHF perivascular deposition.	(23)
Pick's disease	Pick bodies	Microvasculature thinning, increased BV tortuosity, fragmented or twisted capillaries in association with decreased number of long microvessels and their branches.	(149)
Progressive supranuclear palsy	NFT	Tau immunoreactivity in brainstem vasculature.	(147)
Parkinsonism dementia complex of Guam	NFT	Reduced cerebral BV density and ramification, increased vascular fragmentation, and thin capillaries. String and coiling BVs, restricted to the areas affected by NFT.	(40, 150)
Chronic traumatic encephalopathy	NFT	Perivascular NFT in frontal, temporal, and parietal cortices. Astrocytic tangles detected around small cortical BVs.	(148)
Progressive supranuclear palsy	NFT	Cortical tau-positive dense-packed fibrils in astrocytes.	(151–153)
Corticobasal degeneration	NFT	Tau astrocytic plaques in gray and white matter of the cortex, basal ganglia, diencephalon, and rostral brainstem.	(154)
Pick's disease	Pick bodies	Reactive astrocytes containing hyperphosphorylated tau.	(155)

A recent study has demonstrated that primary rat brain ECs (RBEC) exposed to human oligomeric tau dramatically increase the expression of genes related to inflammation, endocytosis, angiogenesis, blood coagulation, and vasoconstriction (142). The authors have also observed upregulated expression of genes involved in diapedesis, the process of immune cell migration across the endothelial wall from the blood circulation to brain parenchyma. Using RBEC monolayers as an *in vitro* BBB model, they have analyzed tau-induced transmigration of peripheral blood monocyte-derived macrophages (PB-MoM) isolated from rat. When RBEC are incubated in conditioned media (CM) of astrocytes previously challenged with tau, a 3-fold accelerated transmigration of PB-MoM has been detected. In the same conditions, EC pre-treatment with monoclonal antibodies anti-ICAM-1 and anti-VCAM-1 reduces PB-MoM transmigration, indicating that these specific endothelial adhesion molecules may play a role in tau-induced trafficking of blood cells across BBB (142). The effects of human truncated tau have been evaluated in a similar *in vitro* model of BBB, consisting of a co-culture of primary RBEC and mixed rat primary astrocyte (85–90%)-microglia (10–15%) cultures (22). When tau is added to the abluminal compartment of the co-culture chamber, where astrocytes and microglia are seeded, a significant decrease of trans-endothelial electrical resistance (TEER) has been observed, concomitantly with an augmented mannitol permeability through the BBB, in contrast to controls. The measurement of pro-inflammatory molecules indicates that BBB breakdown is likely mediated by TNF-α and the chemokine MCP-1, which are abundantly released by glial cells, following tau treatment (22). A different *in vitro* study confirmed the harmful effect of tau on NVU components, showing that cultured rat microglia, transfected with human tau40, were significantly activated compared to control. Activation was confirmed by higher ability of migration, enhanced phagocytosis and increased CM levels of nitric oxide (NO), and IL-1β, IL-6, TNF-α, and IL-10 inflammatory cytokines (145), which may act as mediators of tau-induced BBB dysfunction. Indeed, if on one hand IL-1β and TNF-α regulate the expression of endothelial TJ proteins (156, 157), on the other hand, stimulation of ECs with both TNF-α and IFN-γ increases the level of adhesion molecules, such as ICAM-1 and VCAM-1 (158–161), facilitating immune cell paracellular migration to cerebral parenchyma and amplifying tau-triggered toxicity.

Overall, these *in vitro* studies point to deleterious effects of tau on vascular and immune cellular and molecular pathways responsible for NVU function. These effects and the main respective references are summarized in Table 1.

### Animal Studies

Several *in vivo* studies have contributed to corroborate the impact of tau pathology on the brain vasculature and NVU (Table 1). In one of these studies, as a model of tauopathy, wild-type mice have been injected with recombinant adeno-associated virus (AAV) vector to express mutant tau P301L (encoding for familial FTD tau protein), specifically in neurons (135). Compared to control littermates, AAV-tauP301L–injected animals displayed significantly increased capillary thickness as early as 10 days after injection, with many BVs within the CA1 region of the hippocampus surrounded by swollen astrocytes, most likely participating in the substantial constriction of the capillaries. Interestingly, in these animals, neurodegeneration coincided with microgliosis, which occurred 3–6 weeks following injection, demonstrating that tau-mediated vascular defects preceded neuronal loss. In concomitance with the onset of neurodegeneration, BBB integrity was further compromised, as parenchymal IgGs, IgMs, and α2-macroglobulin were detected (135). In aged (12–15-month-old) tetracycline inducible rTg4510 mice, which overexpress tau P301L protein, a variety of vascular abnormalities have been described, mainly in hippocampus and cortex. Progressively with age, rTg4510 animals exhibited tau perivascular accumulation together with BBB breakdown, measured as significant CD3^+^ and CD4^+^ lymphocyte, and red blood cell (RBC) infiltration along the vasculature, and extravasation of IgGs and Evans Blue, a dye that binds serum albumin, which does not cross the BBB, unless it is injured (24). In this model, tau pathology triggered glial activation, identified as high expression of glial fibrillary acidic protein (GFAP), astrocytic intermediate filament protein, and heat shock protein 27 (Hsp27), marker of reactive astrocytes (162) and involved in BBB regulation through its role in actin stabilization (163). Noteworthy, in comparison to WT or younger Tg animals, treatment with doxycycline, which suppressed tau expression, led to BBB functional recovery in old rTg4510 mice, with reduced T cell and RBC infiltration, and decreased Hsp27 and GFAP levels (24), indicating tau direct involvement in CVD. Transmigration of blood-borne cells, such as PB-MoM, has also been reported in cerebral areas with increased ICAM-1 expression, associated with neurofibrillary pathology, in Tg SHR-72 rat model which stably expresses human tau protein truncated at amino acids 151–391 (aa 151-391/4R) (142), confirming the role of tau in triggering detrimental changes in BBB. Using the Tg4510 murine model, another study has documented multiple brain vascular changes, including increased number of capillaries characterized by atypical and spiraling morphologies, reduced vessel diameter, and elevated cortical BV density, in 15-month-old Tg animals. RNA analysis has revealed an altered expression of hypoxia- and angiogenesis-related genes specifically in ECs and microglia. The greatest fold change has been found in endothelial *Serpine1*, a gene that encodes for plasminogen activator inhibitor (PAI-1) protein (137), known for stimulating migration of ECs (164), modulating proteolytic activity (165), fundamental for extracellular matrix remodeling during angiogenesis, and for regulating microglia motility and phagocytosis (166). Intriguingly, also Tg mice expressing the T34 human tau isoform specifically in astrocytes, have been shown to develop BBB disruption, verified as IgG and albumin presence in the brain parenchyma, in association with prominent tau pathology in astrocytic foot processes surrounding BVs (146). In summary, tau has been shown to trigger multiple detrimental brain vascular and glial changes in animal models, which are detailed in Table 1.

### Human Studies

Over the last few decades, the AD cerebral microvasculature has been described as atrophic, thin, fragmented, twisted or tortuous, and with glomerular loop formations (40). Ultrastructural analysis has reported atrophic ECs, and remarkably decreased (167) and compromised morphology (168) of TJs. In addition, AD brains present anomalous focal constrictions and general degeneration of SMC (169–171), swollen astrocytic end-feet (172, 173), and atrophic pericytes (169). As a consequence, AD subjects display disrupted CBF and brain hypoperfusion (28, 174–176). Interestingly, a 2016 study has demonstrated that vascular changes are correlated with Braak stages, which classify the degree of tau pathology, showing that small and medium size artery SMC loss occurs between early onset tau toxicity-Braak stage I and II–III, along with PHF perivascular deposition (23).

These results bolster a direct connection of tau pathology with vascular abnormalities. As a confirmation, in PiD, where cerebral aggregates of hyperphosphorylated tau (Pick bodies) are present (149), hallmarks like microvasculature thinning, increased BV tortuosity, fragmented or twisted capillaries in association with decreased number of long microvessels and their branches, are as severe as in AD (40). Moreover, atrophic brain areas display a considerable disorganization of the vascular basal lamina distribution (150). A recent paper has described oligomeric tau immunoreactivity in brain vasculature of other tauopathies, such as PSP (147). It is also known that Parkinsonism dementia complex of Guam, characterized by abundant tau neurofibrillary pathology, shows reduced cerebral BV density and ramification, increased vascular fragmentation, and thin capillaries (40). Furthermore, string and coiling vessels are typical, and restricted to the areas affected by NFT (150). Repetitive mild or moderate traumatic brain injury (TBI) seems to be associated with higher risk of AD development (177), and may lead to chronic traumatic encephalopathy (CTE) (148, 178, 179). Strikingly, perivascular NFT of hyperphosphorylated tau are one of the most common pathological hallmarks of CTE, starting at very early stages of the disease, mainly in frontal, temporal, and parietal cortices (particularly in the depth of the sulci) (180). With the progression of the disorder, also fibrillar astrocytic tangles are detected around small cortical BVs (148), plausibly exacerbating vascular dysfunction. Similarly, other tauopathies display high phosphorylated tau accumulation in astrocytes. For instance, in PSP, astroglial phenotype is often referred to as tufted (151–153), showing tau-positive dense-packed fibrils forming tufts around single or double nuclei, mainly in the frontal cortex, striatum, and thalamus (181, 182). Additionally, CBD is characterized by tau astrocytic plaques (154), defined as punctate or spindle-shaped aggregates, forming irregular rounded structures (181, 182). Reactive astrocytes containing hyperphosphorylated tau have been found in PiD as well (155).

Associations between tau pathology and white matter hyperintensities (WMH) have also been reported, showing that increasing cortical P-tau burden independently predicted the severity of WMH, indicating a potentially important role of tau in the pathogenesis of WM damage (183, 184).

Collectively, these human studies provide strong evidence for an association between tau toxicity and vascular dysregulation in multiple tauopathies (summarized in Table 2).

To better dissect how tau pathology may affect cerebrovascular function, we propose below some of the molecular and cellular mechanisms which have been shown to be affected by tau, and may also mediate its effects on the vessel walls.

## TAU and Mitochondrial Dysregulation

### Tau and Mitochondrial Dynamics

Mitochondria are essential organelles for cell survival and death, playing a primary role in energy metabolism and apoptotic processes (185). Balanced mitochondrial fission and fusion dynamics are pivotal events in regulating their shape, size, and number, enabling a correct morphology and distribution, and thus their capacity to meet high energy cellular demands, such as those of brain cells (186–188). A cytosolic guanosine triphosphatase (GTPase), named dynamin-like protein 1 (Drp1), assembling into spiral filaments around mitochondria, interacts with outer membrane proteins, including mitochondrial fission factor (Mff) and fission protein-1 (Fis1), to regulate the division process (189). On the other hand, the interaction of mitochondrial outer membrane proteins, such as the GTPases mitofusin 1 and 2 (Mfn1 and Mfn2), with optic dominant atrophy 1 (Opa1) inner mitochondrial membrane protein, mediates fusion (190). It has been reported that mitochondrial activity and phenotype are directly and tightly modulated by cellular and environmental stimuli (191). Therefore, it is not surprising that mitochondrial dysfunction and aberrant morphology are predominant pathological early features of AD brains (192–194).

Although with some discrepancies, most likely due to different tau isoforms and cell types used, multiple *in vitro* studies have pointed to direct effects of tau on mitochondrial dynamics (Table 3). Both human WT full-length (hTau) and P301L mutated tau isoforms have been shown to promote mitochondrial perinuclear accumulation, a feature of AD brains (203), in animal and cellular models (195, 203). Moreover, in HEK293 cells, hTau enhanced Mfn1, Mfn2, and Opa1 protein expression. Specifically, the decrease of hTau-mediated Mfn2 polyubiquitination underlaid Mfn2 accumulation, and the resulting increased mitochondrial fusion (195). Conversely, primary cortical neurons from tau KO mice, transfected with caspase-3 truncated tau presented mitochondrial fragmentation, together with a significant reduction of Opa1 levels, compared to control neurons (196). Another study has reported that, following treatment with okadaic acid, a well-known PP2A inhibitor, rat brain ECs (RBE4) displayed elevated tau phosphorylation and increased mitochondrial fission (Drp1 and Fis1) and fusion (Mfn1, Mfn2, and Opa1) protein levels, suggesting tau-dependent mitochondrial dynamics alterations, and confirming the expression of tau in ECs (197). Mfn1, Mfn2, and Opa1 accumulation have also been detected in brains of 6-month-old hTau Tg mice, in comparison with age-matched WT littermates (195). Additionally, in tau P301L Tg mice, another *in vivo* model of tauopathy, the partial genetic ablation of Drp1 reduced mitochondrial dysfunction and rescued mitochondrial dynamics (198). Excessive mitochondrial fission triggered by an atypical interaction between hyperphosphorylated tau and Drp1 has been described in multiple AD mouse models (199). Importantly, similar findings have been reported in AD human brains, where phosphorylated tau has been found to physically interact with Drp1, with little or no physical interaction occurring in control subjects (199). Moreover, a disrupted balance of fission and fusion, both at mRNA and protein levels (200, 201), has been found in AD patients, with most studies reporting a shift toward fission (200, 202). Overall, these data point to direct effects of tau on mitochondrial dynamics in multiple cellular (including ECs), animal models of tauopathies, and in human AD brains. These effects and their specific references are summarized in Table 3.

**Table 3:** Tau and mitochondrial dynamics.

**Model/disease**	**Tau isoform**	**Mitochondrial dynamics**	**References**
HEK293 cells and rat primary hippocampal neurons	Human WT full length tau	Mitochondrial perinuclear accumulation. In HEK293 cells, hTau enhances expression of mitochondrial fusion proteins (Mfn1, Mfn2, and Opa1). In HEK293 cells, Mfn2 accumulation is due to hTau-mediated Mfn2 decreased polyubiquitination.	(195)
Primary cortical neurons (from tau KO mice)	Caspase-3 truncated tau (D421)	Mitochondrial fragmentation. Reduction of Opa1 levels.	(196)
RBE4 (rat brain ECs)	WT	Following treatment with okadaic acid (PP2A inhibitor), increased mitochondrial fission (Drp1 and Fis1) and fusion (Mfn1, Mfn2, and Opa1) protein levels.	(197)
Tg hTau mouse	Human WT full length tau	Hippocampal accumulation of Mfn1, Mfn2, and Opa1.	(195)
Tg4510 × Drp1^+/−−^ mouse	P310L tau (neuronal expression)	Drp1 partial genetic ablation decreases Drp1 and Fis1 mRNA and protein levels, in cortical and hippocampal tissues. Drp1 partial genetic ablation increases Mfn1, Mfn2, and Opa1 mRNA and protein levels, in cortical and hippocampal tissues.	(198)
3xTg mouse and Alzheimer's disease	NFT	Excessive mitochondrial fission due to Drp1 and hyperphosphorylated tau interaction.	(199)
Alzheimer's disease	NFT	Disrupted balance of fission and fusion (mRNA and protein), shifted toward fission.	(200–202)

### Tau and Mitochondrial Dysfunction

Mitochondria are important for multiple cell functions in healthy and diseased brains, including energy production, intracellular Ca^2+^ homeostasis control, cell cycle regulation, ROS generation, apoptosis, and, in neurons, synaptic plasticity maintenance (10, 15, 185, 204–209). Amongst these, a pivotal role of the mitochondria is to provide energy to the cell from nutrient sources, through adenosine trisphosphate (ATP) production, accomplished *via* tricarboxylic acid cycle (TCA or Krebs cycle) and oxidative phosphorylation (OxPhos). Mitochondrial bioenergetics dysfunction and elevated mitochondrial ROS production have been reported to cause neuronal degeneration, and eventually cell death, in AD and other neuropathological conditions (194, 205, 206, 210). Mitochondrial dysfunction is also considered one of the earliest and probable causative steps in the AD pathogenesis (211–215). Tau has been recognized as a mediator of mitochondrial dysfunction, in both *in vitro* and *in vivo* models (216–219), as well as in human tauopathies, including AD (Table 4).

**Table 4:** Tau and mitochondrial dysfunction.

**Model/disease**	**Tau isoform**	**Mitochondrial dysfunction**	**References**
RBE4 (rat brain ECs)	WT	Following treatment with okadaic acid (PP2A inhibitor), increased ROS production, mitochondrial Ca^2+^ overload, and activation of caspase-3 and caspase-9.	(197)
Co-culture rat primary neurons + rat primary astrocytes	h4R tau (K18 fragment)	Mitochondrial Ca^2+^ efflux blocked in both neurons and astrocytes.	(220)
Human iPSC-derived cortical neurons	10 + 16 MAPT	Mitochondrial Ca^2+^ overload. Mitochondrial depolarization. Caspase-3 activation.	(220)
Rat primary hippocampal neurons	hTau	Decrease in ATP levels, ATP/ADP ratio and complex I activity.	(195)
SHSY5Y	P301L tau	ATP depletion. Complex I activity deficit and depolarization of MMP.	(219)
Cortical neurons	Caspase-3 truncated tau (D421)	Increased ROS production. Reduction of Ca^2+^-buffering capacity, mitochondrial membrane integrity and MMP. Cyclosporin A (mPTP inhibitor) treatment partially prevents mitochondrial impairment. Mitochondrial fragmentation.	(221)
WT C57BL/6 mouse	Oligomeric tau injection	Following subcortical tau injection, decrease of complex I protein expression, and activation of caspase-9, in the hippocampus.	(218)
Tg4510 mouse	P310L tau (neuronal expression)	Increased amounts of H_2_O_2_ and HNE. Diminished levels of ATP and complex IV, in cortical tissues.	(198)
Tg4510 mouse	P310L tau (neuronal expression)	12-month-old mice display reduced activities of complex I and V. 24-month-old mice show reduced electron transport capacity and ATP levels. Increased H_2_O_2_ and superoxide anion radical levels.	(217)
Frontotemporal dementia	NFT	Complex V level reduction, in temporal cortices.	(217)
Alzheimer's disease	NFT	Cortical complex IV activity is reduced. Reduction of complex I (24- and 75-kDa subunits) and complex V protein levels.	(222–224)
Alzheimer's Disease	NFT	Decreased ATP production. Increased oxidative stress markers (free radicals, lipid peroxidation, and DNA and protein oxidation), in frontal, parietal, and temporal lobes.	(225–228)
Alzheimer's disease	NFT	Mitochondrial structural abnormalities in the vascular wall.	(229)

Cell experiments showed that rat brain ECs challenged with okadaic acid, prompted tau hyperphosphorylation in concomitance with ROS production, mitochondrial Ca^2+^ overload, and activation of caspase-3 and caspase-9, markers of apoptosis (197). The treatment of rat primary neuronal-astrocytic co-culture with the repeat domain of tau (K18) blocked mitochondrial Ca^2+^ efflux *via* impairment of NCLX, mitochondrial Na^+^/Ca^2+^ exchanger, in both neurons and astrocytes. Mitochondrial Ca^2+^ overload occurred also in human iPSC-derived cortical neurons expressing 10 + 16 MAPT mutation (linked to FTD), along with mitochondrial depolarization and caspase-3 activation (220). Rat primary hippocampal neurons stably expressing hTau displayed a significant decrease in ATP levels, ATP/ADP ratio, complex I activity, and cell viability (195). Similarly, overexpression of P301L tau in neuronal SHSY5Y cells led to ATP depletion and pronounced complex I activity deficit, along with depolarization of mitochondrial membrane potential (MMP) (219), which is physiologically generated by complexes I, III, and IV. In cortical neurons, the inducible expression of D421-cleaved caspase-3 truncated tau triggered mitochondrial fragmentation, increased ROS production, and a significant reduction of Ca^2+^-buffering capacity, mitochondrial membrane integrity and MMP. Interestingly, the treatment with cyclosporin A, which inhibits mitochondrial permeability transition pore (mPTP), partially prevented tau-induced mitochondrial impairment (221). mPTP is a mitochondrial channel that opens in pathological circumstances, enhancing mitochondrial permeability to ions and small molecules (230–232), therefore inducing MMP failure, decrease of ATP production, release of mitochondrial content, and cell death (233–237). One of the components of mPTP is voltage-dependent anion channel (VDAC) protein (238). Remarkably, VDAC protein levels have been described to progressively increase in correlation with Braak stages in AD brains, where phosphorylated tau has been found to interact with VDAC (239). WT mice subjected to subcortical injection of tau oligomers exhibited decrease of NADH-ubiquinone oxidoreductase (complex I) protein expression and a considerable activation of caspase-9, when the hemispheres were compared with the ones injected with fibrillar or monomeric tau. Validating the tau propagation hypothesis, these changes have been found in the hippocampus, where IHC analysis revealed the co-localization between tau oligomers and mitochondria, specifically in CA1 cells (218). In another study, P301L mice exhibited increased amounts of hydrogen peroxide (H_2_O_2_) and 4-hydroxy-2-nonenol (HNE), indicator of lipid peroxidation, when compared to WT animals. Moreover, Tg animals showed markedly diminished levels of ATP and cytochrome C oxidase (complex IV) (198), further corroborating the role of tau toxicity in mitochondrial bioenergetics failure. Using the same murine model of tauopathy, it has been demonstrated that brains of 12-month-old Tg mice displayed significantly reduced activities of complex I and V. In these animals, tau-induced mitochondrial dysregulation worsened with aging, since 24-month-old mice showed increasingly reduced electron transport capacity and ATP levels, together with incremented ROS levels (H_2_O_2_ and superoxide anion radicals) (217). Of note, complex V level reduction has been also found in human familial FTD brains (217). In AD, several studies have shown significant decrease of mitochondrial proteins and activity (222–224). Additional studies have observed decreased ATP production and increased oxidative stress markers, such as free radicals, lipid peroxidation, and DNA and protein oxidation (225–228). Strikingly, mitochondrial structural abnormalities have been reported to occur also in the vascular wall of AD subjects, compared to age-matched controls (229). The literature summarized above points to a contribution of tau to mitochondrial dysfunction. Although more studies specifically targeted to understand the mitochondrial effects of tau in ECs or the NVU are still needed, the available data (197) suggest that mitochondrial dysfunction may be one of the mechanisms by which tau impairs cerebrovascular health.

## TAU and Caspases

Caspases are cysteine-dependent proteases which cleave multiple intracellular substrate proteins after an aspartic acid residue, playing a primary role in apoptosis (240–244). Based on their structure and their hierarchical position in the apoptotic signaling cascade, caspases are categorized into upstream initiators (caspase-2,−8,−9, and−10) and downstream effectors (caspase-3,−6, and−7) (245–247). In brain cells, including neurons and glial cells, caspase activation does not always result in apoptosis (248, 249).

In neurodegenerative disorders, including AD, several stressors, such as deficits of oxygen and growth factors, excitotoxicity, inflammation, dysregulation of Ca^2+^ homeostasis and oxidative stress, may induce caspase activation, contributing to the etiopathogenesis of the disease (250–252). The associations between tau toxicity and caspase activation, and the relative references are listed in Table 5.

**Table 5:** Tau and caspases.

**Model/disease**	**Tau isoform**	**Caspase activation**	**References**
Tg4510 mouse	P310L tau (neuronal expression)	Activation of caspase-3 precedes NFT. Following doxycycline treatment (to suppress tau expression), active caspase-3 levels in tangle-bearing neurons are diminished.	(253)
WT mouse	h4R and caspase-3 truncated tau (D421)	h4R-tau injection triggers neuronal caspase-3 activation associated with tau truncation and aggregation. In truncated tau-injected animals, D421^+^ neurons showed colocalization of cleaved tau with the endogenous tau, together with an accumulation of misfolded tau.	(253)
Traumatic brain injury (rat)		Active caspase-3 and D421-cleaved caspase-3 truncated tau presence within GFAP^+^, CD68^+^, and EBA^+^ cells, in the corpus callosum.	(254)
Alzheimer's disease	NFT	Caspase-3,−6,−7,−8, and−9 upregulation, in hippocampus and cortex.	(69, 255–258)
Alzheimer's disease	NFT	Active caspase-3 within NFT in the limbic cortex considered one of the earliest biomarkers of AD.	(259)
Alzheimer's disease	NFT	Active caspase-3 with a high degree of colocalization in neurons, astrocytes and BVs, mainly in hippocampus and entorhinal cortex.	(256)
Alzheimer's disease	NFT	Activation of caspase-3 in CD68^+^ cells (microglia) in the frontal cortex.	(248)
Frontotemporal dementia	NFT	Neurons and degenerating astrocytes positive for active caspase-3, in the temporal lobe.	(260)
Vascular dementia	NFT	Caspase-3 derived tau fragments found in NFT in the hippocampus, DG and frontal cortex. Active caspase-3 (colocalizing with cleaved tau) detected in BVs and pre-tangle neurons.	(261)
Alzheimer's disease	NFT	2–3-fold increase of active caspase-6 in the temporal and frontal cortex. Caspase-6 cleaved tau found into intracellular, extracellular, and even immature tangles.	(257)

Multiple studies have provided evidence that human AD brains display significant caspase-3,−6,−7,−8, and−9 upregulation, compared to controls (69, 255–258). A large body of literature has focused on caspase-3, recognized as the main effector of the apoptotic process (262, 263). The presence of active caspase-3 within NFT is considered one of the earliest biomarkers of AD (259), with a high degree of colocalization in neurons, astrocytes and BVs, in subjects with overt pathology (256). This underpins a tight correlation between tau toxicity and neurovascular dysfunction, which is possibly caspase-mediated. To further corroborate this hypothesis, another study has demonstrated the activation of caspase-3 in CD68^+^ cells, identified by the authors as reactive microglia, in the frontal cortex of AD brains, relative to age- and gender-matched healthy controls (248). Moreover, neurons and a subset of degenerating astrocytes positive for active caspase-3 have been described in FTD cerebral samples (260). The activation of caspase-3 has been described to precede and to lead NFT formation in 7-month-old rTg4510 mice. Remarkably, when animals have been treated with doxycycline to suppress tau expression, the levels of cleaved caspase-3 in tangle-bearing neurons were diminished, while tangles remained, suggesting that soluble tau is the upstream initiator of caspase-3 activation, which in turn leads to the fibrillary progression (253).

Interestingly, among more than 400 proteins that can be cleaved by caspases (264–266), tau has been detected as substrate of caspase-2 (267),−3 (65), and−6 (69). Tau fragments derived from caspase-3 and−6 cleavage have been found in AD subjects (65, 69). Specifically, D421-cleaved caspase-3 truncated tau is accounted as an early feature of AD (268), and it has also been observed in other tauopathies, including PiD, PSP and CBD (269). Considering that caspase-3 cleaved tau shows an enhanced propensity to phosphorylation (65), and therefore to self-aggregation, besides the canonical role in apoptosis, caspase-3 activation may also function as a trigger for tau polymerization and NFT assembly (45, 47, 59, 66), ultimately causing dendritic spine loss and synaptic dysfunction (67, 68), as well as possible toxic effects for neurovascular cells. Accordingly, the injection of human tau-4R in WT mice led to the neuronal activation of caspase-3, tau truncation and aggregation. Moreover, when animals were injected with truncated tau, D421^+^ neurons showed colocalization of cleaved tau with the endogenous WT isoform, together with an accumulation of misfolded tau, further confirming that D421 tau fragment precedes and is sufficient to induce tau conformational and phosphorylation changes, and that the process is likely caspase-3-mediated (and initiated by soluble tau) (253). Active caspase-3 and D421-cleaved caspase-3 truncated tau have also been reported to occur within GFAP^+^, CD68^+^, and EBA^+^ (endothelial barrier antigen) cells in the white matter of corpus callosum, in a rat model of TBI, corroborating the involvement of caspase-3 activation in tau-mediated neurovascular damage (254). In addition, caspase-3 derived tau fragments have been found in NFT in the hippocampus, DG and frontal cortex, in human vascular dementia brains. Importantly, active caspase-3 has been detected in BVs and pre-tangle (but not NFT) neurons that colocalized with cleaved tau, supporting that caspase-3 activation is a required step for the cleavage of tau and the consequential NFT development at the NVU level (261).

Since Aβ oligomeric aggregates have also been shown to induce caspase-3 activation in neurovascular cells, including endothelial, glial, and neuronal cells (10, 13–15, 206–208), the presence of Aβ deposits surrounding cerebral vessels in AD and CAA, promoting caspase-3 activation, may also prompt an increase in the levels of caspase-3 truncated tau, which is more prone to phosphorylation and aggregation, and may therefore enhance tau toxicity at the NVU (270). In addition, AD subjects display a 2–3-fold increase of active caspase-6 in the temporal and frontal cortex, where caspase-6 cleaved tau is found into intracellular, extracellular, and even immature tangles (257).

Overall, this evidence supports the hypothesis that both tau and Aβ can initiate caspase activation, which may lead to apoptosis and, concomitantly, promote tau aggregation and NFT formation, further exacerbating tau-induced neurovascular pathology. In particular, the presence of active caspases in the brain vasculature (254, 256, 261), accompanied by the discussed evidence of tau transmission within neurovascular cells, supports the hypothesis that caspase activation may induce accumulation of toxic caspase-3 cleaved tau in cerebrovascular and glial cells, thus precipitating neurovascular pathology in AD, CAA, and tauopathies.

## Discussion

The impact of tau on neurovascular pathology, although previously understudied, has recently become an active topic of research for the AD and dementia scientific community. Although more specific mechanistic studies in vessel wall cells are needed, here we postulate that tau may propagate from neurons to NVU cellular components such as ECs, astrocytes and microglia, and that inflammatory and mitochondrial alterations induced by tau in these cells may underlie its toxicity at the NVU (Figure 1). We further hypothesize that caspase activation, and in particular active caspase-3 tau cleavage, may also play a primary role in pathological tau-induced vascular dysfunction. Whether oligomeric Aβ aggregates, possibly through caspase-mediated mechanisms, contribute to perivascular tau oligomerization and NFT deposition is an intriguing hypothesis in need of additional exploration. Contextually, the lack of knowledge of the cell-specific detrimental molecular mechanisms initiated by tau arises new questions. Further studies will be necessary to elucidate tau-driven endothelial, pericyte, astrocytic, and microglial harmful cellular events. Amongst these, it will be critical to dissect the possible link between tau dysregulated clearance, altered inflammatory response and development of tau aggregates around cerebral BVs, in neurodegenerative diseases, including AD and other tauopathies, such as PiD, PSP, and CTE. Although tau toxicity seems to be responsible for neurovascular compromise, other studies suggest that early CVD can also cause tau pathological accumulation (136). Both these mechanisms may induce a vicious cycle between CVD and tau pathology, which still remains largely understudied. Establishing causality of these effects when studying a disease like AD, presenting with different pathologies, such as amyloidosis, tauopathy, cerebrovascular dysfunction, and neuroinflammation, often overlapping at the same time in the same brains, will require multiple and properly-designed studies.

Moreover, developing strategies to promote perivascular clearance, such as immunotherapy (271) and to target neurovascular cell-specific mechanisms, including mitochondrial dysfunction (207, 208, 272), to counteract toxicity of both amyloid and tau, will be essential research efforts to ameliorate CVD in AD and other tauopathies.

## Author Contributions

EC and SF designed and conceptualized the review. EC wrote the review draft and did the literature search. SF critically revised and edited the manuscript, provided relevant insights, additional literature search, and acquired funding. Both authors contributed to the article and approved the submitted version.

## Conflict of Interest

The authors declare that the research was conducted in the absence of any commercial or financial relationships that could be construed as a potential conflict of interest.
